# Modern Sociology

**Published:** 1903-09-12

**Authors:** 


### Modern Sociology.

### THE SIMPLER LIFE.

We hail with pleasure the issue of a sixpenny edition of Dr. George Keith's "Plea for a Simpler Life." This little book, written with so much sincerity and in such good style, may be safely commended for popular reading. The public are addressed on matters medical from many sides. First we have the newspapers, which are seldom without items of medical interest relating to the public health from week to week, to the innumerable congresses, to new discoveries, announced by telegraph or otherwise, to the various "campaigns" which are in progress. Next we have the omnipresent quack, whose advertisements may take the form of a succinct exposition of the principles of health and disease, or of a recital of symptoms in authentic cases relieved by his particular nostrum. Or, lastly, we have the old-established books and articles on domestic medicine and hygiene, which are usually an echo of current professional teaching. But it is not often that the laity are invited to follow the arguments and counsels of an original thinker on medicine whose message is worth listening to. The great essayists of the profession, Sir Thomas Browne, Wendell Holmes, or Dr. John Brown, have given us hardly more of medicine proper than Montaigne himself. They are philosophers at large, who would have spoiled their style and would have fallen under the suspicion of "talking shop" if they had applied their reflective and musing genius to their proper business. Yet there is plenty of room for corresponding paradoxes, quaint reflections, and genial philosophic maxims upon diseases and cures. What little wit the profession now spends on its proper themes is apt to turn to satire upon the endless ramifications of the microbic theory, or upon the latest fashions in practice, both surgical and medical. Or, if we come upon a serious essayist with some claim to distinction, such as the author of the "Simpler Life," we find him to be an enthusiast moved to deliver himself of a particular idea.

Dr. Keith may not profess to be a pathologist and he is probably innocent of any learned contribution to the science, but there is found underlying his views of regimen and treatment a deep principle concerning the nature of disease which is the philosophic outcome of his experience and reflection. Disease is not the original evil, but rather "the means adopted by nature for the cure, or the checking, of some evil lurking in the system." Only a few lines are given to the development of this promising paradox. It is clearly something more than Charcot's famous phrase, that symptoms are "the cry of the suffering organs." It means that morbid processes, which may become, as we all know, hideous enough and malignant, are in their proper nature conservative efforts of the organ or part to carry on its work under some stress or strain, making a bold fight for self-preservation, struggling to the last by means of every ingenious device, but, of course, struggling hopelessly in very many cases. "Some evil lurking in the system" is the occasion of all those reactions, adaptations, or makeshifts which we call disease. However, it appears from the practical sequel that the evil does not always "lurk." When one saturates the system with alcohol the primary evil is far from recondite. It is rather the efforts to save itself which are made by the poor body thus misused that are difficult to follow. The connection between cause and effect is not always so exquisitely simple as in Marryat's tale of the bargee's wife, who was so saturated with gin that she took fire and burned to a cinder; or in the more elaborately related case by Dickens, which the great novelist wrote a whole preface to defend. But, such dramatic effects apart, many diseases are claimed to be of the nature of an answer made by the body to the various abuses that it is put to; and the simpler life is the way to stop the abuses. We eat too much, we drink too much, we smoke too much, and we err in a dozen other ways. Moreover—and this is the author's great heresy—we make a mistake in trying to correct the mischief by means of drugs, or even by a sojourn at a spa. Such, in a bare outline, is the philosophy of the simpler life in so far as it touches the health of the body.

The simpler life has been led by many in all ages, and by not a few sects bound by a common rule. Those of a certain temperament or disposition will be satisfied with the minimum of self-indulgence—with "food to keep us till we end our mortal span and the pure goal of being reach." But the ambition of the average sensual man is to have a good time; and he looks to the profession of medicine to patch up his old body, if not for heaven, as was said of Jack Falstaff's, yet for as long a continuance upon earth as may be compatible with a liberal enjoyment of the good creatures of life. There are deliberate philosophies and creeds of the ample life as well as of the meagre. There is a whole science and art of gastronomics, which is not altogether indifferent to dietetic principles. Again, there are the alleged millions who cannot always be sure of having enough to satisfy the natural craving of hunger. To one large class the counsels of a simpler life are not admissible on the principles of the epicure; to another still larger class they are superfluous, so far at least as concerns any danger of plethora. But there are always many individual cases to whom the starving system will appeal; such as the two men, in a single day, who were converted by Dr. Keith's amiable talk in the smoking-room of an Atlantic liner. We cordially wish him many more converts. But, although his teaching is hardly eclectic in the form which he has given to it, yet it is necessarily eclectic in its successful working. The proverb says, with as much truth as proverbs usually have, that one man's meat is another man's poison. Not only so, but granting that one has been using too full a diet of food and beverages, it becomes a nice question whether "the evil lurking in the system" as a result thereof can be met by a change to abstinence. Habit must always be reckoned with, whether in the literal sense of what we have been used to or in the derived sense of constitution. A famous surgeon, who had gout and lived to an advanced age, once laid down the specious principle to a fellow sufferer: "You and I have got it, and what we have to do is to live up to it." On the other hand, the author of this plea is himself an authentic instance of the benefits of a simpler life began after middle age; and another eminent authority, Sir Henry Thompson, lays down the principle that the latter half of life rather admits of abstemiousness than needs keeping up. It is certainly suprising to the routine diner to find how little food suffices for some who have tried the experiment. The late Sir Erskine May, clerk of the Parliament, who "looked more like a healthy English farmer," told Dr. Keith that his rule was, while the House was sitting, to take a chop in the middle of the day, and only a cup of tea at night; and he added that Lord Palmerston followed the same plan, though on occasion he could enjoy a very full dinner. While the author is a somewhat rigid teacher, he is agreeably free from fanaticism; and as his professed aim is to stir the laity to think for themselves in such matters, he may be congratulated upon the aids to reflection which his genial writing supplies.

